# A Multidimensional Bayesian Network Meta-Analysis of Chinese Herbal Injections for Treating Non-small Cell Lung Cancer With Gemcitabine and Cisplatin

**DOI:** 10.3389/fphar.2021.739673

**Published:** 2021-09-06

**Authors:** Mengwei Ni, Zhishan Wu, Haojia Wang, Wei Zhou, Chao Wu, Antony Stalin, Changgeng Fu, Peizhi Ye, Shan Lu, Yingying Tan, Zhihong Huang, Xiaotian Fan, Jingyuan Zhang, Xiaomeng Zhang, Miaomiao Wang, Jiarui Wu

**Affiliations:** ^1^ Department of Clinical Chinese Pharmacy, School of Chinese Materia Medica, Beijing University of Chinese Medicine, Beijing, China; ^2^ State Key Laboratory of Subtropical Silviculture, Department of Traditional Chinese Medicine, Zhejiang A&F University, Hangzhou, China; ^3^ Xiyuan Hospital of China Academy of Chinese Medical Sciences, Beijing, China; ^4^ National Cancer Center/National Clinical Research Center for Cancer/Chinese Medicine Department of the Cancer Hospital of the Chinese Academy of Medical Sciences and Peking Union Medical College, Beijing, China

**Keywords:** non-small cell lung cancer, network meta-analysis, chinese herbal injections, gemcitabine plus cisplatin, multidimensional cluster

## Abstract

**Introduction:** As non-small cell lung cancer (NSCLC) seriously threatens human health, several clinical studies have reported that Chinese herbal injections (CHIs) in combination with and gemcitabine plus cisplatin (GP) are beneficial. This multidimensional network meta-analysis aimed to compare the clinical efficacy and safety of different CHIs in combination with GP against NSCLC.

**Methods:** Randomized controlled trials (RCTs) for the treatment of NSCLC were retrieved from seven electronic databases from inception to April 30, 2020. Study selection and data extraction were based on a priori criteria. Data analysis was performed using Stata 13.0, WinBUGS 14.0 software. Multidimensional cluster analysis was performed using the “scatterplot3d” package in R 3.6.1 software.

**Results:** This network meta-analysis included 71 eligible RCTs and 10 Chinese herbal injections. Delisheng injection and Kangai injection had the highest probability in terms of clinical effectiveness rate (94.60%) and gastrointestinal reactions (82.62%) when combined with GP compared with the other interventions. Compound Kushen injection combined with GP ranked ahead of the other interventions in terms of performance status (73.36%) and abnormal liver function (87.17%). Shenmai injection combined with GP had the highest probability in terms of leukopenia (94.59%) and thrombocytopenia (99.18%).

**Conclusion:** The current evidence revealed that CHIs combined with GP have a better impact on patients with NSCLC than GP alone. Aidi injection, Compound kushen injection, and Kanglaite injection deserve more attention of clinicians when combined with GP in patients with NSCLC. Additionally, due to the limitations of this network meta-analysis, further well-designed, large-sample, multicenter RCTs are required to support our findings adequately.

## Introduction

According to the International Agency for Research on Cancer (Sung et al., 2021), lung cancer is the second most commonly diagnosed cancer. It is the leading cause of cancer death, accounting for 11.4 and 18.0% of new cases and deaths of all cancers worldwide. With a 5-year survival rate of only 10–20% in most countries (Allemani et al., 2018), lung cancer has a poor prognosis. Based on histological type, lung cancer is internationally divided into small-cell lung cancer and non-small-cell lung cancer (NSCLC), with NSCLC accounting for approximately 80% of all lung cancer cases (Siegel et al., 2021). The primary treatment for early-stage lung cancer is surgery. However, due to the insidious nature of lung cancer and the delay in seeking medical treatment, 50% of patients missed the best time for surgery at diagnosis (La Fleur et al., 2019). Primary treatments for patients who cannot undergo surgery include targeted therapies, immunotherapies, and the combination of two drugs based on platinum (Maione et al., 2011; Ettinger et al., 2021), such as gemcitabine plus cisplatin (GP), paclitaxel plus cisplatin and vinorelbine plus cisplatin. GP regimen is commonly used for NSCLC patients, and its antineoplastic efficacy has been endorsed. However, this treatment is associated with a highly toxic physiological environment and adverse events that may even lead to treatment discontinuation (Lischalk et al., 2016; Osarogiagbon et al., 2016).

**FIGURE 1:**
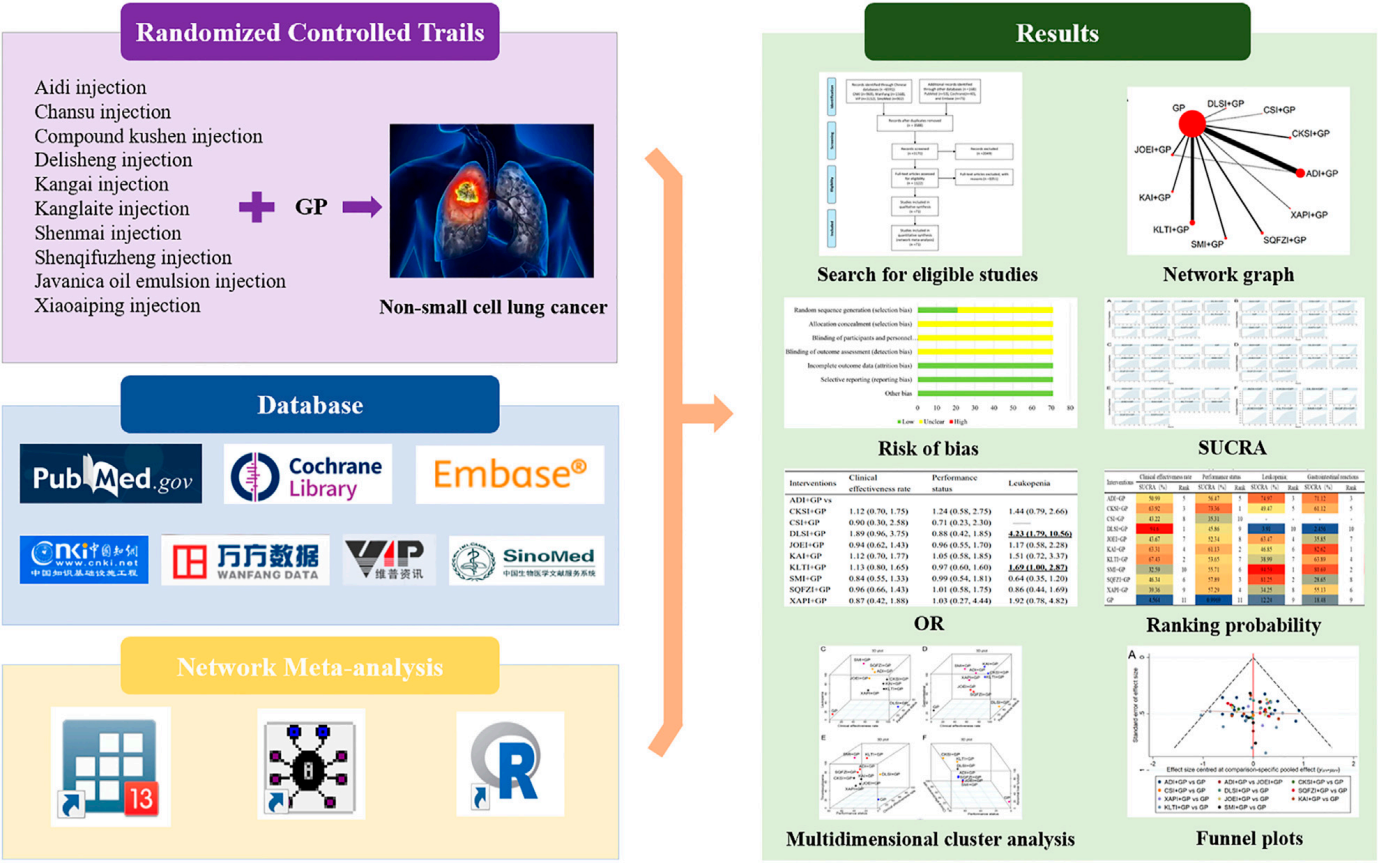
Graphical abstract of the network meta-analysis. Note: GP, gemcitabine plus cisplatin; SUCRA, surface under the cumulative ranking curve; OR, odds ratio.

As a complementary and alternative medicine, traditional Chinese medicine (TCM) has gradually gained acceptance as an adjuvant treatment for cancer (Bao et al., 2014; Xiang et al., 2019). Studies have shown that TCM has a positive impact in retarding cancer progression and ameliorating chemotherapy-induced complications and adverse events (Hofseth and Wargovich, 2007; Konkimalla and Efferth, 2008). TCM in lung cancer, especially in advanced lung cancer, has accumulated rich clinical experience that can improve patients' quality of life and long-term survival (Chen et al., 1999). Chinese Herbal Injections (CHIs) are an indispensable part of TCM. Chinese patent medicines have been used most frequently in lung cancer patients, and CHIs account for the most significant proportion of Chinese patent medicines (Wu et al., 2015). There have been various CHIs used in the treatment of NSCLC, and the optimal strategy for combining CHIs with GP to treat NSCLC remains unclear. Therefore, this study systematically evaluates the efficacy of 10 CHIs in combination with GP for the treatment of NSCLC through a network meta-analysis (NMA) to provide evidence-based medicine for clinicians to choose an optimal strategy. The graphical abstract of this NMA is shown in Figure 1.

## Methods

This study was conducted in accordance with the Preferred Reporting Items for Systematic Reviews and Meta-Analyses (PRISMA) Extension Statement for Reporting of Systematic Reviews Incorporating Network Meta-analyses of Health Care Interventions (Hutton et al., 2015). A completed PRISMA checklist is included in Supplementary File S1.

### Eligibility Criteria and Exclusion Criteria

Randomized controlled trials (RCTs) of CHIs in combination with GP for the treatment of NACLC. The article describes that “random” can be included, and the blind was unrestricted. The study included patients with TNM stage Ⅲ or Ⅳ NSCLC diagnosed by cytology or pathology, with no restrictions on gender, age, race, region or nationality. Interventions with any Chinese herbal injection combined with GP to treat NSCLC. The control group included GP alone OR any other Chinese herbal injection. There were no limitations on the dosages or treatment courses. If patients had complications during the therapeutic process, the corresponding therapy had to be adopted.

The exclusion criteria were as follows: 1) patients had any other primary tumor; 2) interventions included surgery or other Chinese medicine treatments, such as other Chinese patent medicine, Chinese herbal decoction, acupuncture, and massage; 3) duplicate literature, we included the first, larger sample size, and more information publication; 4) did not report relevant outcomes or corresponding criteria for evaluating the efficacy; 5) non-intravenous administration of CHIs; 6) self-controlled studies or randomized methods are high-risk such as alternation, assignment based on date of birth, case record number and date of presentation and 7) unclear drug name, dosages, or treatment courses.

The primary efficacy outcome was the clinical effective rate. The secondary outcome was performance status, and the adverse drug reactions (ADRs) outcomes were leukopenia, gastrointestinal reactions, thrombocytopenia, and abnormal liver function. 1) Clinical effectiveness rate. According to the response evaluation criteria in solid tumors by the WHO, the clinical effectiveness rate can be divided into four levels: complete response (CR), in which visible lesions disappear completely after >4 weeks; partial response (PR), in which the tumor area of a single lesion has been reduced by ≥50% or the sum of the products of the two largest vertical diameters of multiple lesions has been reduced by >50%; stable disease (SD), in which no significant change occurred within at least 4 weeks and estimated tumor size increased by <25% or decreased by <50%; and progressive disease (PD), in which new lesions or original lesions increased by ≥25%. The following formula calculated the clinical effectiveness rate: clinical effectiveness rate = (number of CR patients + number of PR patients)/total number of patients × 100%. 2) Performance status. The performance status was evaluated by the Karnofsky performance status (KPS) score. An increase or decrease in KPS score by <10 points was considered stable after treatment; increase in KPS score by ≥10 points was considered to improvement performance status after treatment; while decrease in KPS score by ≥10 points was considered to reduce in the performance status. Performance improvement rate = the number of patients with improved performance/total number of patients × 100%. 3) leukopenia, gastrointestinal reactions, thrombocytopenia, and abnormal liver function. They were evaluated according to the WHO criteria for acute and subacute toxic reaction of anticancer drugs formulated in 1981. The ADRs were divided into 5 grades. The incidence of ADRs = number of patients with ADRs/total number of patients × 100%.

### Search Strategy

Seven electronic databases, including PubMed, Cochrane Library, Embase, Chinese National Knowledge Infrastructure, Chinese Biomedical Literature Database, Chinese Scientific Journals Full-text Database, and Wanfang Database, were searched from inception to April 30, 2020. To obtain the relevant literature, articles were retrieved by the combination of medical subject heading (MeSH) and free-text keywords, and the search strategies were constructed for three domains: 1) NSCLC, 2) CHIs, and 3) study type (RCTs). Using PubMed as an example, the following terms were used for NSCLC: “Non-Small-Cell Lung Carcinomas [MeSH Terms],” “Non-Small-Cell Lung Carcinoma,” “Nonsmall Cell Lung Cancer,” “Non Small Cell Lung Carcinoma,” “Non-Small Cell Lung Carcinoma,” and “Non-Small Cell Lung Cancer.” In addition, there were no restrictions on the publication year or language. Details on the retrieval strategies are provided in Supplementary File S2.

### Data Extraction

After deleting duplicate records by NoteExpress software (Wuhan University Library, Wuhan, China), two investigators independently screened the titles to remove the obviously irrelevant studies such as reviews and experimental animal reports. They also read the abstracts and full texts of the remaining studies to screen for potential studies according to the inclusion criteria and extracted data from eligible RCTs. Any divergences were resolved through discussion or by the third reviewer in the implementation process.

The following data were collected according to the predesigned form. 1) Publication information: Title, first author's name, and publication year. 2) Characteristics of the enrolled patients with NSCLC: sample size, age, gender, pathological type, stage of cancer, and KPS score. 3) Information on the intervention: dosage, duration, and treatment cycle. 4) Outcomes: clinical effectiveness rate, performance status, ADRs such as gastrointestinal reactions and abnormal liver function and immune function related ADRs (leukopenia, thrombocytopenia). 5) Description of study design: blinding, randomized allocation methods, and other quality assessment items.

### Risk of Bias Assessment

Two researchers independently assessed the risk of bias within each study using the Cochrane Risk of Bias Tool recommended by the Cochrane Handbook 5.1 (Higgins et al., 2011). The following domains were assessed: selection bias (random sequence generation and allocation concealment), performance bias (blinding of the participants and personnel), detection bias (blinding of the outcome assessment), attrition bias (incomplete outcome data), reporting bias (selective reporting) and other bias. Each bias has three levels: “low risk”, “unclear risk” and “high risk”. Any discrepancies during this process were resolved either by consensus or by consultation with a third investigator.

### Statistical Analysis

STATA 13.1 software (Stata Corp, College Station, TX, United States) and WinBUGS 1.4.3 software (Medical Research Council Biostatistics Unit, Cambridge, United Kingdom) was used for statistical analysis. All graphs of the NMA were presented using Stata 13.1 software and the thickness of the lines in the network graph was proportional to the number of trials used for the comparisons, and the node sizes corresponded to the total sample sizes for the treatments (Chaimani et al., 2013; Shim et al., 2017). The odds ratio (OR) and its 95% confidence intervals (95% CI) were used to describe the effect for dichotomous outcomes. If the 95% CI did not contain 1, differences between compared groups were statistically significant. The NMA was performed using the WinBUGS software, whereas the Markov chain Monte Carlo method with a random-effects model was used for Bayesian inference. In the WinBUGS software, the number of simulation iterations was 200,000, and the first 10,000 iterations were used for burn-in to eliminate the impact of the initial value (Crainiceanu and Goldsmith, 2010). Moreover, the results of the WinBUGS software calculations were used by the Stata software to calculate the surface under the cumulative ranking curve (SUCRA), which ranged from 0 to 100%. An intervention with a larger SUCRA value was considered the more effective treatment (Rücker and Schwarzer, 2015; Trinquart et al., 2016). The results of the WinBUGS software calculations were employed by the Stata software to obtain SUCRA. Cluster analysis was also performed to comprehensively compare the effect of CHIs on two different outcomes, with the interventions that were located in the upper-right corner being superior to the others. Publication bias was described via a comparison-adjusted funnel plot using Stata software (Trinquart et al., 2012). Symmetric points on the graph indicate that there is no obvious publication bias. Cluster analysis was also performed to compare the effect of CHIs on two different outcomes comprehensively, with the interventions that were in the upper-right corner were superior to the others (Veroniki et al., 2015).

The amount and source of heterogeneity among included RCTs were conducted by Stata 13.0 software that conduct a meta-analysis of direct comparison between CHI+GP and GP analysis for each outcome. The heterogeneity within each injection subgroup was analyzed by Q test, and the *p*-value was used to evaluate the degree of heterogeneity. When *p* > 0.05, the difference within a group is considered small and the heterogeneity is not obvious. For outcomes with obvious heterogeneity, covariates that may have an impact on heterogeneity were analyzed in meta-regression by the restricted maximum likelihood (REML) method to estimate the variance component τ^2^ between studies. Sensitivity analysis investigates the influence of each individual study on the overall meta-analysis summary estimate, and assessed robustness of results. The presence and degree of inconsistency in each loop were infer by using the magnitude of the inconsistency factors (IF), and it was regarded as a better consistency when the lower bound of 95% CIs was equal to zero (Chaimani et al., 2013).

### Multidimensional Cluster Analysis

For a comprehensive assessment of efficacy, multidimensional cluster analysis based on the SUCRA values of any three outcomes of different CHIs was performed using the “scatterplot3d” package in R 3.6.1 software (Mathsoft, Cambridge, United States). The K-means method was used to cluster these interventions, and the number of clusters was modified according to the actual situation. The steps of clustering were as follows: 1) All interventions were randomly divided into k initial classes, and the average of the outcome indicators of these k classes was used as initial aggregation points. 2) An intervention was classified into the closest aggregation point category, and then the category aggregation points were updated to the average of the current outcome indicators. All interventions were recategorized and classified, and step 2) was repeated until all interventions were assigned. Finally, the ranking of interventions with three outcome indicators was visually represented by a three-dimensional stereogram. Different colors were applied to indicate interventions belonging to different categories.

## Results

### Search Results

**FIGURE 2:**
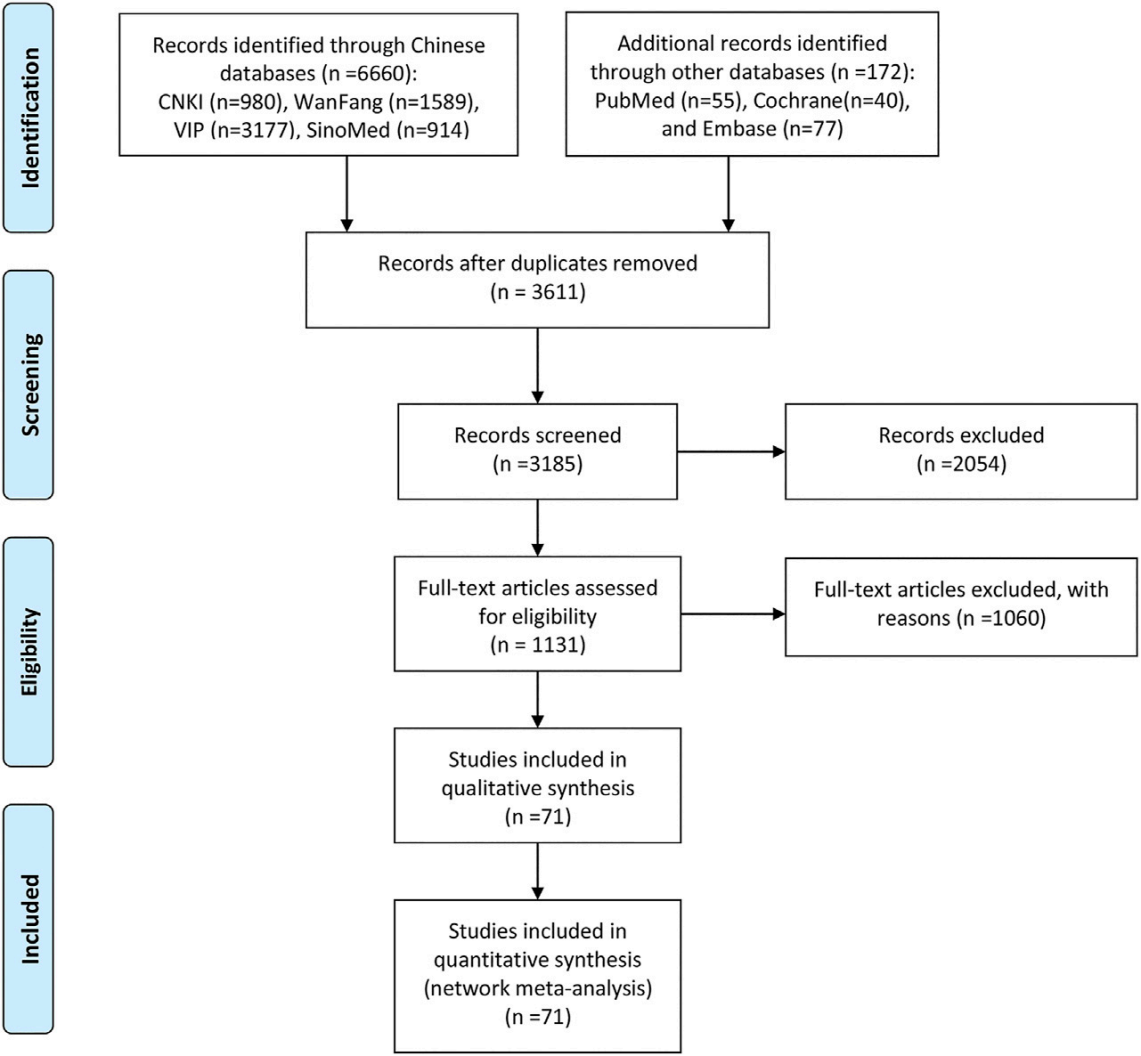
Flow chart of the search for eligible studies. Note: n, number of articles. CNKI, China National Knowledge Infrastructure; WanFang, the WanFang Database; VIP, the Chinese Scientific Journals Full-Text Database; and SinoMed, the Chinese Biomedical Literature Database.

The search strategy initially yielded 6832 prospective articles from the electronic databases. After excluding 3611 duplicates and screening titles and abstracts, 1131 articles remained for further evaluation. After a detailed review, a total of 71 RCTs with 10 CHIs met our selection criteria. The number of included studies for the different CHIs was as follows: Aidi injection (23 RCTs), Chansu injection (1 RCT), Compound kushen injection (5 RCTs), Delisheng injection (2 RCTs), Javanica oil emulsion injection (6 RCTs), Kangai injection (5 RCTs), Kanglaite injection (14 RCTs), Shenmai injection (6 RCTs), Shenqifuzheng injection (7 RCTs), and Xiaoaiping injection (2 RCTs). Information about the included injections is shown in Supplementary File S3. The PRISMA flow diagram of study selection is shown in Figure 2.

### Inclusion Studies and Characteristics

**FIGURE 3:**
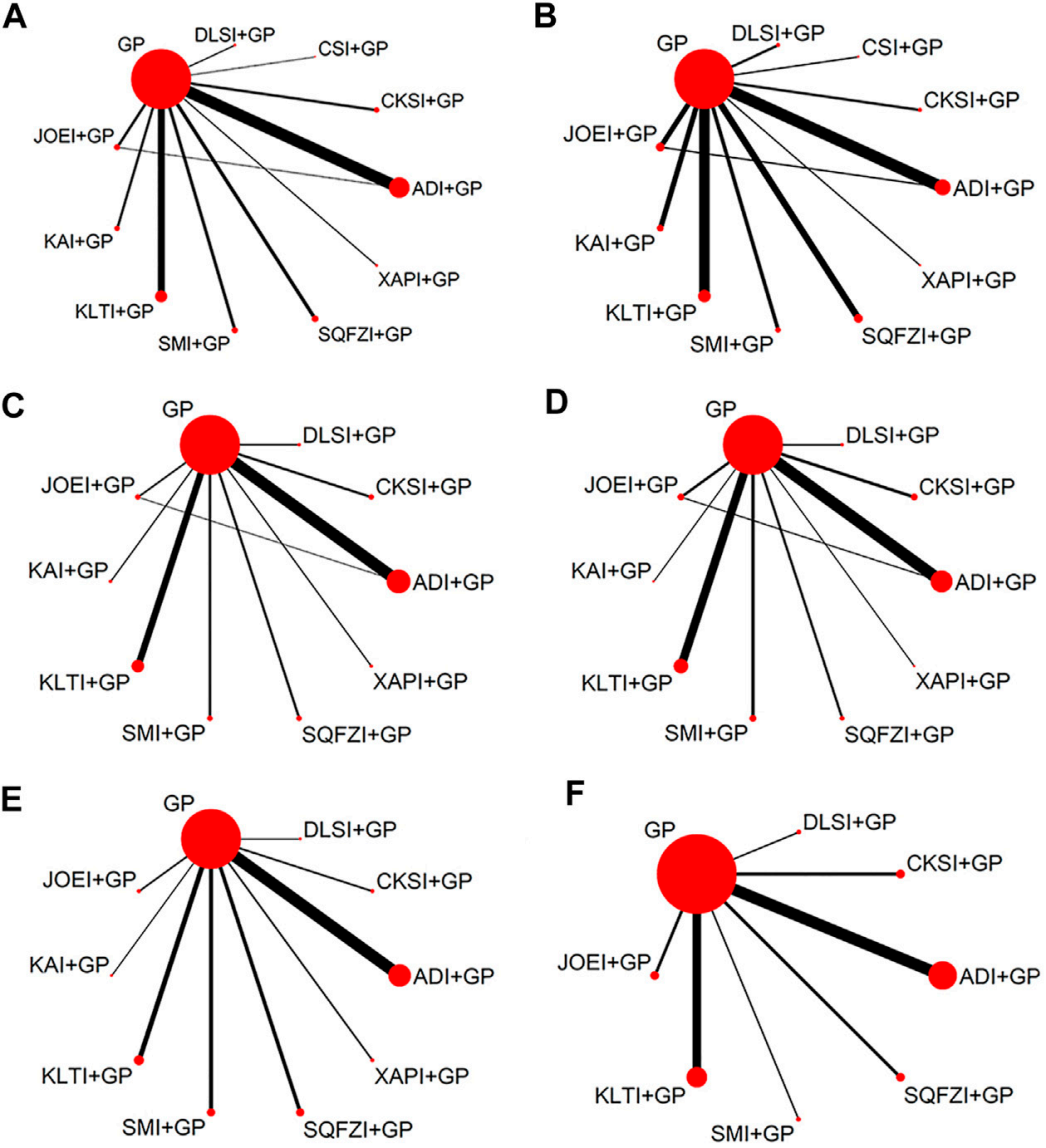
Network graph for different outcomes. **(A)** Clinical effectiveness rate; **(B)** Performance status; **(C)** Leukopenia; **(D)** Gastrointestinal reactions; **(E)** Thrombocytopenia; and **(F)** Abnormal liver function. Note: ADI, Aidi injection; CKSI, Compound Kushen injection; DLSI, Delisheng injection; GP, Gemcitabine plus Cisplatin; JOEI, Javanica oil emulsion injection; KAI, Kangai injection; KLTI, Kanglaite injection; SMI, Shenmai injection; SQFZI, Shenqifuzheng injection; and XAPI, Xiaoaiping injection.

In total, the 71 RCTs enrolled 5728 patients with NSCLC involving 10 CHIs; 2903 of them received a combination of CHIs and GP in the experimental group, and 2825 patients received the only GP in the control group. All RCTs reported the sample size, patients’ age, sex, tumor node metastasis (TNM) stage, and Karnofsky performance status score before treatment. Sixty-nine (97.18%) studies reported the clinical effectiveness rate, and 40 (56.34%), 50 (70.42%), 43 (60.56%), 33 (46.48%), and 20 (28.17%) RCTs reported the performance status, leukopenia, gastrointestinal reactions, thrombocytopenia and abnormal liver function, respectively. Baseline characteristics of all included RCT are summarized in Table 1. The network graphs of the 10 CHIs with different outcomes are depicted in Figure 3.

**TABLE 1:** Characteristics of the included randomized controlled trials.

Study ID	Sex (M/F)	Case (E/C)	Average age (Year)	KPS score	TNM stage	Pathological type	Treatment of experiment	Treatment of control	Course (day)	Outcomes
Liu and Zhao, (2014)	53/33	43/43	39–70/42–73	≥60	Ⅲb, Ⅳ	LAC, LSCC	ADI 50 ml + GCB 1000 mg/m^2^ + DDP 50 mg/m^2^	GCB 1000 mg/m^2^ + DDP 50 mg/m^2^	(8–10/21) × (2)	①②③
Zhang, (2012)	63/20	41/42	57.2 ± 9.4/58.2 ± 10.3	≥60	Ⅲb, Ⅳ	LAC, LSCC, LASC	ADI 60 ml + GCB 1000 mg/m^2^ + DDP 25 mg/m^2^	GCB 1000 mg/m^2^ + DDP 25 mg/m^2^	(14/21) × (3)	①②③
Huang et al. (2017)	46/33	39/40	50–70/49–68	≥60	Ⅲb, Ⅳ	LAC, LSCC, LASC, LCLC	ADI 60 ml + GCB 1000 mg/m^2^ + DDP 25 mg/m^2^	GCB 1000 mg/m^2^ + DDP 25 mg/m^2^	21 × 3	①③④
Xu et al. (2013)	55/25	38/42	39–79/42–81	NR	Ⅲb, Ⅳ	LAC, LSCC	ADI 50 ml + GCB 1000 mg/m^2^ + DDP 30 mg/m^2^	GCB 1000 mg/m^2^ + DDP 30 mg/m^2^	14 × 3	①②③④
Hong et al. (2010)	82/78	90/70	38–70	≥60	Ⅲb, Ⅳ	LAC, LSCC	ADI 60 ml + GCB 1000 mg/m^2^ + DDP 25 mg/m^2^	GCB 1000 mg/m^2^ + DDP 25 mg/m^2^	14/ (21 × 2)	①②③
Ju et al. (2013)	36/32	34/34	61–81/61–78	NR	Ⅲb, Ⅳ	LAC, LSCC, LASC	ADI 50 ml + GCB 1000 mg/m^2^ + DDP 50 mg/m^2^	GCB 1000 mg/m^2^ + DDP 50 mg/m^2^	(14/21) × (2)	①②
Ma, (2017)	55/29	42/42	46–75/44–73	≥60	Ⅲb, Ⅳ	LAC, LSCC, LASC	ADI 50 ml + GCB 1000 mg/m^2^ + DDP 75 mg/m^2^	GCB 1000 mg/m^2^ + DDP 75 mg/m^2^	(21/28) × (4)	①④
He et al. (2011)	29/23	29/23	21–73/29–74	≥60	Ⅲb, Ⅳ	LAC, LSCC	ADI 50–100 ml + GCB 1000 mg/m^2^ + DDP 75 mg/m^2^	GCB 1000 mg/m^2^ + DDP 75 mg/m^2^	(15/21) × (2–3)	①②③
Zhang, (2009)	44/20	32/31	31–77	≥60	Ⅲb, Ⅳ	LAC, LSCC, LCLC	ADI 80 ml + GCB 1000 mg/m^2^ + DDP 80 mg/m^2^	GCB 1000 mg/m^2^ + DDP 80 mg/m^2^	(14/21) × (2)	①②③⑤
Li et al. (2016)	52/42	47/47	40–70/42–67	≥60	Ⅲb, Ⅳ	LAC, LSCC, LASC	ADI 50–100 ml + GCB 1000 mg/m^2^ + DDP 30 mg/m^2^	GCB 1000 mg/m^2^ + DDP 30 mg/m^2^	14/ (28×3–4)	①②③④
Sun et al. (2008)	54/9	33/30	34–73/35–72	≥60	Ⅲb, Ⅳ	LAC, LSCC, LASC	ADI 10 g + GCB 1000 mg/m^2^ + DDP 30 mg/m^2^	GCB 1000 mg/m^2^ + DDP 30 mg/m^2^	(15/21) × (2)	①②③
Xu, (2012)	37/19	28/28	41–75/42–74	NR	Ⅳ	NR	ADI 100 ml + GCB 1000 mg/m^2^ + DDP 30 mg/m^2^	GCB 1000 mg/m^2^ + DDP 30 mg/m^2^	(8/21) × (2)	①②④
Chen and Chen, (2013)	102/34	68/68	31–69	NR	Ⅲb, Ⅳ	LAC, LSCC	ADI 50 ml + GCB 100 mg/m^2^ + DDP 30 mg/m^2^	GCB 100 mg/m^2^ + DDP 30 mg/m^2^	(14/21) × (2)	①③
Jin et al. (2004)	51/17	34/34	29–72	70–90	Ⅲb, Ⅳ	LAC, LSCC	ADI 50 ml + GCB 100 mg/m^2^ + DDP 30 mg/m^2^	GCB 100 mg/m^2^ + DDP 30 mg/m^2^	(14/21) × (2)	①③
Pan et al. (2011)	54/25	41/38	39–71/41–73	≥60	Ⅲb, Ⅳ	LAC, LSCC	ADI 50 ml + GCB 1000 mg/m^2^ + DDP 30 mg/m^2^	GCB 1000 mg/m^2^ + DDP 30 mg/m^2^	(21/21) × (2–4)	①②
Chen et al. (2012)	25/13	19/19	33–71/35–72	≥60	III, IV	LAC, LSCC	ADI 50 ml + GCB 1000 mg/m^2^+ DDP 75 mg /m^2^	JOEI 30 ml + GCB 1000 mg/m^2^ + DDP 75 mg /m^2^	(14/21) × (4)	②③④
Feng et al. (2008)	88/42	68/62	38–70/40–74	70–90	III, IV	LAC, LSCC	ADI 50 ml + GCB 1000 mg/m^2^ + DDP 75 mg/m^2^	GCB 1000 mg/m^2^ + DDP 75 mg/m^2^	30/ (21 × 2)	①②③④
Wen et al. (2009)	52/24	38/38	32–77	NR	III, IV	LAC, LSCC	ADI 50 ml + GCB 1000 mg/m^2^ + DDP 75 mg/m^2^	GCB 1000 mg/m^2^ + DDP 75 mg/m^2^	(8–10/21) × (2)	①②③
Liu et al. (2010)	37/27	32/32	45–73/47–75	>60	III, IV	LAC, LSCC, LCLC, O	ADI 50 ml + GCB 1000 mg/m^2^ + DDP 30 mg/m^2^	GCB 1000 mg/m^2^ + DDP 30 mg/m^2^	(14/14) × (4)	①③
Chen, (2016)	36/24	30/30	42–76	>60	III, IV	LAC, LSCC	ADI 50 ml + GCB 1000 mg/m^2^ + DDP 25 mg/m^2^	GCB 1000 mg/m^2^ + DDP 25 mg/m^2^	8/ (21 × 2)	①④
Wu and Chen, (2017)	83/52	67/68	43–69	NR	III, IV	LAC, LSCC	ADI 100 ml + GCB 1000 mg/m^2^ + DDP 20 mg/m^2^	GCB 1000 mg/m^2^ + DDP 20 mg/m^2^	(10/21) × (4)	①②③
Wang, (2009)	48/11	32/27	NR	≥60	III, IV	NR	ADI + GCB 1000 mg/m^2^ + DDP 20 mg	GCB 1000 mg/m^2^ + DDP 20 mg	(10/21) × (2)	①③
Li et al. (2010)	39/33	36/36	29–75/32–73	>60	III, IV	LAC, LSCC	ADI 50–100 ml + GCB 1000 mg/m^2^ + DDP 30 mg/m^2^	GCB 1000 mg/m^2^ + DDP 30 mg/m^2^	(15/21) × (2)	①②③
Li and Li, (2010)	58/27	43/42	60–82/56–81	≥60	Ⅳ	LAC, LSCC	CKSI 20 ml + GEM 1.4 g + DDP 40 mg	GEM 1.4 g + DDP 40 mg	14/ (28 × 2)	①③
Shan, (2015)	53/37	45/45	60–82	≥60	Ⅲb, Ⅳ	LSCC, LAC, O	CKSI 15 ml + GEM 800 mg/m^2^ + DDP 60 mg/m^2^	GEM 800 mg/m^2^ + DDP 60 mg/m^2^	14/ (21 × 2)	①③
Lu, (2017)	68/52	60/60	50–75/52–73	>70	Ⅳ	LAC, LSCC, LASC, O	CKSI 20 ml + GEM 1000 mg/m^2^ + DDP 20 mg/m^2^	GEM 1000 mg/m^2^ + DDP 20 mg/m^2^	(14/21) × 4	①②③
Song, (2014)	40/20	30/30	43–77/41–75	≥60	Ⅲb, Ⅳ	LAC, LSCC	CKSI 20 ml + GEM 1000 mg/m^2^ + DDP 25 mg/m^2^	GEM 1000 mg/m^2^ + DDP 25 mg/m^2^	21 × 2	①②③
Zhao et al. (2012)	61/25	43/43	50–78/52–76	≥60	Ⅲa, Ⅲb, Ⅳ	LAC, LSCC, LASC, LCLC	CKSI 12 ml + GEM 1000 mg/m^2^ + DDP 70 mg/m^2^	GEM 1000 mg/m^2^ + DDP 70 mg/m^2^	(14/21) × 2	①②
Chen, (2009)	46/16	31/31	56.5	≥60	Ⅲb, Ⅳ	LAC, LSCC, LASC, O	CSI 20 ml + GCB 1000 mg/m^2^ + DDP 80 mg/m^2^	GCB 1000 mg/m^2^ + DDP 80 mg/m^2^	(15–20) ×2	①②③④
He, (2009)	82/39	61/60	35–74/36–73	≥60	IIIb, IV	LAC, LSCC	DLSI 15–20m1 + GEM 1000 mg/m^2^ + DDP 30 mg/m^2^	GEM 1000 mg/m^2^ + DDP 30 mg/m^2^	(14/21) × (3–4)	①②③
Zhou and Ni, (2009)	35/25	30/30	40–72	≥60	Ⅲb, Ⅳ	LAC, LSCC	DLSI 40 ml + GEM 1000 mg/m^2^ + DDP 30 mg/m^2^	GEM 1000 mg/m^2^ + DDP 30 mg/m^2^	14 × 2	①②③④
Tian and Wang, (2017)	63/33	48/48	40–79/42–81	≥60	Ⅲ, Ⅳ	LAC, LSCC, O	JOEI 30 ml + GEM 1000 mg/m^2^ + DDP 25 mg/m^2^	GEM 1000 mg/m^2^ + DDP 25 mg/m^2^	(15/21) × 2	①②
Su, (2017)	42/15	29/28	58.7 ± 7.8/59.7 ± 10.1	≥60	Ⅲb, Ⅳ	LAC, LSCC, O	JOEI 30 ml + GEM1000 mg/m^2^ + DDP 80 mg/m^2^	GEM1000 mg/m^2^ + DDP 80 mg/m^2^	(10/21) × 2	①②③
Chen et al. (2010)	62/24	45/41	38–71/40–70	60–85	Ⅲb, Ⅳ	LAC, LSCC, LASC	JOEI 30–40 ml + GEM 1000 mg/m^2^ + DDP 25 mg/m^2^	GEM 1000 mg/m^2^ + DDP 25 mg/m^2^	21 × 4	①②③
Tong et al. (2011)	33/30	33/30	39–74/52–76	≥60	Ⅲb, Ⅳ	LAC, LSCC	JOEI 30 ml + GEM 1000 mg/m^2^ +DDP 75 mg/m^2^	GEM 1000 mg/m^2^ +DDP 75 mg/m^2^	21 × 2	①②③
Zhang et al. (2017a)	54/24	39/39	67 ± 11/68 ± 12	NR	Ⅲb, Ⅳ	LAC, LSCC, O	JOEI 40 ml + GEM 1000 mg/m^2^ + DDP 30 mg/m^2^	GEM 1000 mg/m^2^ + DDP 30 mg/m^2^	14/ (21 × 2)	①②④
Tian et al. (2007)	79/36	58/57	43–74/42–78	NR	Ⅲa, Ⅲb, Ⅳ	LAC, LSCC, LCLC, O	JOEI 40 ml + GEM 1000 mg/m^2^ + DDP 20 mg/m^2^	GEM 1000 mg/m^2^ + DDP 20 mg/m^2^	14 × 2/21 × 2	①②④
Cui et al. (2012)	46/13	30/29	29–75	≥70	Ⅲb, Ⅳ	LAC, LSCC	KAI 50 ml + GCB 1000 mg/m^2^ + DDP 25 mg/m^2^	GCB 1000 mg/m^2^ + DDP 25 mg/m^2^	21 × 2	①②③
Liang et al. (2009)	51/16	34/33	53.5/51.6	≥50	Ⅲb, Ⅳ	LAC, LSCC, LASC, LCLC	KAI 50 ml + GCB 1000 mg/m^2^ + DDP 80 mg/m^2^	GCB 1000 mg/m^2^ + DDP 80 mg/m^2^	(14/21) × 2	①
Zhou, (2014)	44/36	40/40	62–76	≥60	Ⅲb, Ⅳ	NR	KAI 40 ml + GCB 1200 mg/m^2^ + DDP 30 mg/m^2^	GCB 1200 mg/m^2^ + DDP 30 mg/m^2^	(14 × 4)/ (21 × 4)	①②④
Su, (2010)	96/64	80/70	26–75/23–72	NR	Ⅲ, Ⅳ	LAC, LSCC, LASC, LCLC	KAI 50 ml + GCB 1250 mg/m^2^ + DDP 25 mg/m^2^	GCB 1250 mg/m^2^ + DDP 25 mg/m^2^	(10/21) × 2	①②③④
Yi et al. (2011)	47/17	32/32	70–79/71–78	≥60	Ⅲ, Ⅳ	LAC, LSCC, LASC	KAI 30 ml + GCB 1250 mg/m^2^ + DDP 25 mg/m^2^	GCB 1250 mg/m^2^ + DDP 25 mg/m^2^	21 × 2	①②
Yao and Song, (2017)	78/59	70/67	64.8 ± 7.2/63.4 ± 7.0	>60	Ⅲb, Ⅳ	LAC, LSCC	KLTI 200 ml + GCB 1000 mg/m^2^ + DDP 30 mg/m^2^	GCB 1000 mg/m^2^ + DDP 30 mg/m^2^	14/ (21 × 2)	①③④
Liang et al. (2014)	37/11	25/23	60–75/60–73	NR	Ⅲb, Ⅳ	LAC, LSCC	KLTI 100 ml + GCB 1000 mg/m^2^ + DDP 30 mg/m^2^	GCB 1000 mg/m^2^ + DDP 30 mg/m^2^	21 × 2	①②③
Guan et al. (2010)	40/25	35/30	70–79/70–82	>60	Ⅲb, Ⅳ	LAC, LSCC	KLTI 200 ml + GCB 1000 mg/m^2^ + DDP 25 mg/m^2^	GCB 1000 mg/m^2^ + DDP 25 mg/m^2^	21 × 2	①②③④
Guan et al. (2009)	11/13	12/12	57/55	≥70	Ⅲb, Ⅳ	LAC, LSCC	KLTI 300 ml + GCB 1250 mg/m^2^ + DDP 75 mg/m^2^	GCB 1250 mg/m^2^ + DDP 75 mg/m^2^	21 × 2	①②③
Chen, (2018)	59/43	51/51	57–79/57–79	NR	Ⅲ, Ⅳ	LAC, LSCC, LASC, LCLC	KLTI 200 ml + GCB 1000 mg/m^2^ + DDP 25 mg/m^2^	GCB 1000 mg/m^2^ + DDP 25 mg/m^2^	14/ (21 × 4)	①②③
Tong, (2007)	41/19	30/30	29–67	≥70	Ⅲ, Ⅳ	LAC, LSCC, LASC	KLTI 100 ml + GCB 1000 mg/m^2^ + DDP 25 mg/m^2^	GCB 1000 mg/m^2^ + DDP 25 mg/m^2^	21 × 2	①②③
Zou et al. (2011)	33/16	25/24	67–79	≥70	Ⅲ, Ⅳ	LAC, LSCC	KLTI 200 ml + GCB 1250 mg/m^2^ + DDP 30 mg/m^2^	GCB 1250 mg/m^2^ + DDP 30 mg/m^2^	21 × 2	④
Lu, (2016)	69/55	62/62	30–65/31–64	NR	Ⅲ, Ⅳ	NR	KLTI 100 ml + GCB 800 mg/m^2^ + DDP 80 mg/m^2^	GCB 800 mg/m^2^ + DDP 80 mg/m^2^	21 × 3	①②
Liu et al. (2017)	43/29	34/38	66/64	NR	Ⅲ, Ⅳ	NR	KLTI 20 g + GCB 800 mg/m^2^ + DDP 30 mg/m^2^	GCB 800 mg/m^2^ + DDP 30 mg/m^2^	8/ (21 × 2)	①②
Wang et al. (2015)	51/29	40/40	52–68/52–69	NR	Ⅲb, Ⅳ	LAC, LSCC, LASC	KLTI 200 ml + GCB 1000 mg/m^2^ + DDP 80 mg/m^2^	GCB 1000 mg/m^2^ + DDP 80 mg/m^2^	10 × 6	①②③
Liu, (2016)	35/25	30/30	55–78/52–72	NR	Ⅲ, Ⅳ	LAC, LSCC, LASC	KLTI 100 ml + GCB 1000 mg/m^2^ + DDP 25 mg/m^2^	GCB 1000 mg/m^2^ + DDP 25 mg/m^2^	21 × 4	①②③
Zhu and You, (2016)	49/36	43/42	48–73/56–80	≥60	Ⅲ, Ⅳ	LAC, LSCC, LASC	KLTI 200 ml + GCB 1250 mg/m^2^ + DDP 25 mg/m^2^	GCB 1250 mg/m^2^ + DDP 25 mg/m^2^	10/ (21 × 2)	①③④
You and Jia, (2011)	23/5	14/14	37–75 /41–73	≥60	Ⅲ, Ⅳ	NR	KLTI 100 ml/d + GCB 1000 mg/m^2^ + DDP 30 mg/m^2^	GCB 1000mg/m^2^ + DDP 30 mg/m^2^	21 × 4	①②③
Wang et al. (2017)	32/40	36/36	52.14 ± 2.01/52.42 ± 2.11	NR	Ⅲ, Ⅳ	LAC, LSCC	KLTI 60 ml + GCB 1250 mg/m^2^ + DDP 75 mg/m^2^	GCB 1250 mg/m^2^ + DDP 75 mg/m^2^	7 × 4	①②③
Li et al. (2013)	70/37	54/53	38–76/41–79	≥60	Ⅲb, Ⅳ	LAC, LSCC, LASC, O	SMI 50 ml + GEM 1000 mg/m^2^ + DDP25 mg/m^2^	GEM 1000 mg/m^2^ + DDP25 mg/m^2^	(10/21) × 2	①②③
Shang et al. (2012)	35/15	25/25	42–77/44–75	>60	Ⅲb, Ⅳ	LAC, LSCC, LASC, O	SMI 50 ml + GEM 1000 mg/m^2^ + DDP25 mg/m^2^	GEM 1000 mg/m^2^ + DDP25 mg/m^2^	21 × 2	①
Qiu, (2013)	21/9	15/15	41–75	>60	Ⅲb, Ⅳ	NR	SMI 50 ml + GEM 1000 mg/m^2^ + DDP 25 mg/m^2^	GEM 1000 mg/m^2^ + DDP 25 mg/m^2^	10/ (21 × 2)	①②③
Li et al. (2012)	35/15	25/25	42–77/44–75	≥60	Ⅲb, Ⅳ	LAC, LSCC, LASC, O	SMI 50 ml + GEM 1000 mg/m^2^ + DDP 25 mg/m^2^	GEM 1000 mg/m^2^ + DDP 25 mg/m^2^	21 × 2	①③
Fu et al. (2012)	42/18	30/30	37–79	60–90	Ⅲa, Ⅲb, Ⅳ	LAC, LSCC	SMI 60 ml + GEM 1000 mg/m^2^ + DDP 25 mg/m^2^	GEM 1000 mg/m^2^ + DDP 25 mg/m^2^	(15/21) × 3	①②③
Feng et al. (2009)	90/75	85/80	33–75	≥60	Ⅲ, Ⅳ	LAC, LSCC	SMI 100 ml/d + GEM 1000 mg/m^2^ + DDP 75 mg/m^2^	GEM 1000mg/m^2^ + DDP 75 mg/m^2^	(15 × 2)/ (21 × 2)	①②③④
Zhou et al. (2009)	43/27	35/35	34–70	>60	Ⅲb, Ⅳ	LAC, LSCC, LCLC	SQFZI 250 ml + GCB 1250 mg/m^2^ + DDP 25 mg/m^2^	GCB 1250 mg/m^2^ + DDP 25 mg/m^2^	(10/21) × 4	①②③④
Yao et al. (2013)	84/16	50/50	31–70/30–70	>70	Ⅲ, Ⅳ	LAC, LSCC	SQFZI 250 ml + GCB 1000 mg/m^2^ + DDP 50 mg/m^2^	GCB 1000 mg/m^2^ + DDP 50 mg/m^2^	21 × 2	①②③
Huang et al. (2014)	51/25	38/38	56.84 ± 10.31/57.28 ± 9.28	>70	Ⅲb, Ⅳ	NR	SQFZI 250 ml + GCB 1000 mg/m^2^ + DDP 100 mg/m^2^	GCB 1000 mg/m^2^ + DDP 100 mg/m^2^	7/ (21 × 2)	①②③
An and Cao, (2014)	49/48	52/45	39–73/40–76	≥60	Ⅲb, Ⅳ	LAC, LSCC, LCLC	SQFZI 250 ml + GCB 1000 mg/m^2^ + DDP 30 mg/m^2^	GCB 1000 mg/m^2^ + DDP 30 mg/m^2^	(14/28) × 2	①③
Zhao, (2014)	62/40	51/51	46–77/48–76	≥60	Ⅲb, Ⅳ	LAC, LSCC, LASC	SQFZI 250 ml + GCB 1000 mg/m^2^ + DDP 25 mg/m^2^	GCB 1000 mg/m^2^ + DDP 25 mg/m^2^	21 × 2	①④
Gui and Tian, (2016)	64/29	45/48	36–74/39–75	NR	Ⅲ, Ⅳ	NR	SQFZI 260 ml + GCB 1500 mg/m^2^ + DDP 70 mg/m^2^	GCB 1500 mg/m^2^ + DDP 70 mg/m^2^	(10/21) × 4	①③
Zhang et al. (2017b)	59/45	52/52	41–82/42–81	NR	Ⅲb, Ⅳ	NR	SQFZI 250 ml + GCB 1000 mg/m^2^ + DDP 25 mg/m^2^	GCB 1000 mg/m^2^ + DDP 25 mg/m^2^	10/ (21 × 2)	①④
Fang et al. (2013)	56/30	43/43	41–86	>70	Ⅲb, Ⅳ	LAC, LSCC, LCLC	XAPI 2 ml + GEM 1000 mg/m^2^ + DDP 30 mg/m^2^	GEM 1000 mg/m^2^ + DDP 30 mg/m^2^	21 × 3	①②③
Zhang et al. (2011)	31/17	24/24	50–75	>50	Ⅲb, Ⅳ	LAC, LSCC	XAPI 40–60 ml + GEM 1000 mg/m^2^ + DDP 30 mg/m^2^	GEM 1000 mg/m^2^ + DDP 30 mg/m^2^	15/ (21 × 2)	①②③④

Note: ADI, Aidi injection; C, Control group; CKSI, Compound Kushen injection; CSI, Chansu injection; DDP, Cisplatin; DLSI, Delisheng injection; E, Experimental group; GEM, Gemcitabine; JOEI, Javanica oil emulsion; KAI, Kangai injection; KLTI, Kanglaite injection; NR, Not reported; SMI, Shenmai injection; SQFZI, Shenqifuzheng injection; XAPI, Xiaoaiping injection; ①, Clinical effectiveness rate; ②, Performance status; ③, Adverse reactions; ④, Immune function.

### Risk of Bias Assessment

**FIGURE 4:**
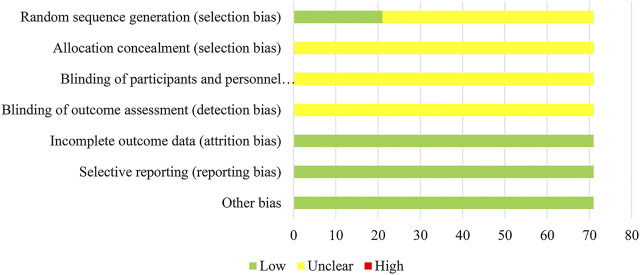
Assessment of risk bias.

In terms of random sequence generation, 21 of 71 studies used reasonable methods to generate the random sequence, 16 RCTs (22.54%) used a random number table and 5 RCTs (7.04%) used a lottery; thus, the selection bias of these studies was rated as “low risk”. Other studies were rated as “unclear risk”. None of the included studies provided information on the allocation concealment and blinding methods, so performance bias and detection bias were assessed as “unclear”. In addition, as all RCTs had no incomplete data and selective reporting, their attrition bias and reporting bias were evaluated as “low risk”. For the assessment of the quality of the RCTs with regard to other bias, the original studies did not report inconsistent baselines, or other problems associated with high risk of bias; therefore, the other bias was noted as “low risk”. The details of the risk of bias assessment for all included studies are shown in Figure 4.

### Network Meta-analysis

This study conducted subgroup analysis with direct comparison and sensitivity on the involved six outcomes. The two outcomes, leukopenia, and thrombocytopenia, with significant heterogeneity in the subgroup analysis were further analyzed by meta regression. Then two factors of DDP dose and cancer grade were explored relationship with heterogeneity. The tumor stages included in this study were all stage Ⅲ-Ⅳ, which were consistent and had no obvious clinical heterogeneity. Therefore, this study only performed regression analysis on DDP dose. The regression results showed that dosage was not a statistically significant source of heterogeneity (*p* > 0.05). Sensitivity investigates the results of two outcomes in the two models were similar, and the data was steady. The details were shown in the Supplementary File S4.

The consistency test was performed for the outcome of clinical effectiveness rate. This NMA involved one triangular loop. The result (IF = 0.175, 95% CI =0.00–1.53) indicated that there is no evidence of significant inconsistency.

#### Clinical Effectiveness Rate

A total of 69 studies with 10 CHIs and 11 interventions reported clinical effectiveness rates in the NMA. The results suggested 7 types of CHIs: Aidi injection, Compound kushen injection, Delisheng injection, Javanica oil emulsion injection, Kangai injection, Kanglaite injection, and Shenqifuzheng injection, combined with GP were significantly more effective than GP alone. Combined with GP, Delisheng injection might have a greater potential to increase the clinical effectiveness rate than Shenmai injection. The network graph is depicted in Figure 3A, and the ORs with 95% CI are presented in Table 2.

**TABLE 2:** Odds ratio/mean difference (95%CI) of all therapeutic interventions.

Interventions	Clinical effectiveness rate	Performance status	Leukopenia	Gastrointestinal reactions	Thrombocytopenia	Abnormal liver function
ADI+GP vs						
CKSI+GP	1.12 (0.70, 1.75)	1.24 (0.58, 2.75)	1.44 (0.79, 2.66)	1.13 (0.65, 1.95)	1.49 (0.63, 3.49)	0.44 (0.16, 1.15)
CSI+GP	0.90 (0.30, 2.58)	0.71 (0.23, 2.30)	——	——	——	——
DLSI+GP	1.89 (0.96, 3.75)	0.88 (0.42, 1.85)	**4.23 (1.79, 10.56)**	**4.13 (1.96, 8.95)**	1.70 (0.50, 5.91)	1.27 (0.34, 4.60)
JOEI+GP	0.94 (0.62, 1.43)	0.96 (0.55, 1.70)	1.17 (0.58, 2.28)	1.68 (0.89, 3.19)	1.59 (0.66, 3.75)	1.08 (0.33, 3.51)
KAI+GP	1.12 (0.70, 1.77)	1.05 (0.58, 1.85)	1.51 (0.72, 3.37)	0.79 (0.34, 1.80)	1.59 (0.46, 5.78)	——
KLTI+GP	1.13 (0.80, 1.65)	0.97 (0.60, 1.60)	**1.69 (1.00, 2.87)**	1.08 (0.69, 1.71)	0.70 (0.35, 1.38)	0.59 (0.21, 1.56)
SMI+GP	0.84 (0.55, 1.33)	0.99 (0.54, 1.81)	0.64 (0.35, 1.20)	0.87 (0.51, 1.46)	**0.33 (0.15, 0.67)**	1.03 (0.10, 8.26)
SQFZI+GP	0.96 (0.66, 1.43)	1.01 (0.58, 1.75)	0.86 (0.44, 1.69)	1.94 (0.93, 4.09)	1.06 (0.53, 2.10)	0.99 (0.31, 3.04)
XAPI+GP	0.87 (0.42, 1.88)	1.03 (0.27, 4.44)	1.92 (0.78, 4.82)	1.23 (0.22, 5.83)	1.67 (0.61, 4.35)	——
GP	**0.60 (0.49, 0.74)**	**0.31 (0.22, 0.42)**	**2.80 (2.16, 3.69)**	**2.27 (1.75, 2.94)**	**1.98 (1.40, 2.86)**	**1.70 (1.06, 2.70)**
CKSI+GP vs						
CSI+GP	0.80 (0.25, 2.51)	0.57 (0.16, 2.19)	——	——	——	——
DLSI+GP	1.70 (0.77, 3.68)	0.71 (0.27, 1.90)	**2.92 (1.10, 8.19)**	**3.66 (1.55, 8.93)**	1.15 (0.28, 4.77)	2.88 (0.64, 13.21)
JOEI+GP	0.84 (0.49, 1.49)	0.78 (0.33, 1.77)	0.81 (0.34, 1.86)	1.49 (0.70, 3.22)	1.06 (0.36, 3.24)	2.49 (0.61, 10.20)
KAI+GP	1.00 (0.56, 1.79)	0.84 (0.35, 1.95)	1.05 (0.44, 2.64)	0.70 (0.28, 1.73)	1.08 (0.26, 4.42)	——
KLTI+GP	1.02 (0.62, 1.65)	0.78 (0.35, 1.74)	1.18 (0.57, 2.35)	0.96 (0.52, 1.78)	0.47 (0.18, 1.23)	1.35 (0.39, 4.74)
SMI+GP	0.76 (0.43, 1.34)	0.80 (0.33, 1.89)	**0.45 (0.20, 0.98)**	0.77 (0.40, 1.49)	**0.22 (0.08, 0.59)**	2.37 (0.20, 21.73)
SQFZI+GP	0.86 (0.51, 1.46)	0.81 (0.35, 1.90)	0.60 (0.26, 1.36)	1.72 (0.74, 3.88)	0.72 (0.26, 1.87)	2.22 (0.59, 9.38)
XAPI+GP	0.78 (0.34, 1.83)	0.82 (0.19, 4.06)	1.34 (0.48, 3.71)	1.10 (0.19, 5.52)	1.12 (0.34, 3.61)	——
GP	**0.54 (0.36, 0.81)**	**0.25 (0.12, 0.50)**	**1.94 (1.13, 3.36)**	**2.01 (1.25, 3.24)**	1.33 (0.62, 2.89)	**3.82 (1.66, 9.69)**
CSI+GP vs						
DLSI+GP	2.13 (0.62, 7.22)	1.24 (0.35, 4.30)	——	——	——	——
JOEI+GP	1.05 (0.34, 3.25)	1.35 (0.40, 4.40)	——	——	——	——
KAI+GP	1.24 (0.41, 3.91)	1.49 (0.42, 4.69)	——	——	——	——
KLTI+GP	1.27 (0.42, 3.84)	1.38 (0.43, 4.20)	——	——	——	——
SMI+GP	0.94 (0.30, 2.98)	1.4 0(0.42, 4.45)	——	——	——	——
SQFZI+GP	1.08 (0.35, 3.33)	1.42 (0.45, 4.51)	——	——	——	——
XAPI+GP	0.98 (0.28, 3.54)	1.46 (0.25, 9.35)	——	——	——	——
GP	0.67 (0.23, 1.96)	0.44 (0.15, 1.25)	——	——	——	——
DLSI+GP vs						
JOEI+GP	0.50 (0.23, 1.05)	1.09 (0.48, 2.47)	**0.28 (0.09, 0.79)**	0.4 (0.16, 1.01)	0.93 (0.23, 3.82)	0.86 (0.17, 4.38)
KAI+GP	0.59 (0.28, 1.28)	1.19 (0.52, 2.66)	0.36 (0.12, 1.09)	**0.19 (0.06, 0.53)**	0.93 (0.17, 5.41)	——
KLTI+GP	0.60 (0.30, 1.19)	1.10 (0.51, 2.44)	0.40(0.15, 1.03)	**0.26 (0.11, 0.58)**	0.41 (0.11, 1.53)	0.46 (0.10, 2.05)
SMI+GP	**0.45 (0.21, 0.94)**	1.12 (0.49, 2.60)	**0.15 (0.05, 0.41)**	**0.21 (0.09, 0.49)**	**0.19 (0.05, 0.72)**	0.81 (0.06, 8.96)
SQFZI+GP	0.51 (0.24, 1.04)	1.16 (0.51, 2.50)	**0.20 (0.07, 0.57)**	0.46 (0.17, 1.25)	0.63 (0.16, 2.29)	0.78 (0.16, 3.83)
XAPI+GP	0.46 (0.18, 1.27)	1.17 (0.26, 5.71)	0.45 (0.13, 1.50)	0.29 (0.05, 1.64)	0.97 (0.23, 4.26)	——
GP	**0.32 (0.17, 0.61** )	**0.35 (0.18, 0.69)**	0.66 (0.28, 1.51)	0.55 (0.26, 1.09)	1.17 (0.35, 3.79)	1.33 (0.4, 4.52)
JOEI+GP vs						
KAI+GP	1.19 (0.68, 2.06)	1.09 (0.55, 2.16)	1.30 (0.51, 3.52)	0.47 (0.17, 1.25)	1.00 (0.24, 4.42)	——
KLTI+GP	1.21 (0.74, 1.95)	1.02 (0.54, 1.88)	1.45 (0.67, 3.23)	0.64 (0.32, 1.31)	0.44 (0.16, 1.15)	0.54 (0.13, 2.23)
SMI+GP	0.90 (0.52, 1.56)	1.03 (0.51, 2.05)	0.56 (0.23, 1.33)	0.52 (0.24, 1.09)	**0.20 (0.07, 0.56)**	0.95 (0.07, 9.84)
SQFZI+GP	1.02 (0.62, 1.71)	1.06 (0.54, 2.01)	0.74 (0.30, 1.82)	1.15 (0.47, 2.85)	0.67 (0.25, 1.76)	0.91 (0.20, 3.94)
XAPI+GP	0.93 (0.41, 2.14)	1.08 (0.26, 4.78)	1.65 (0.56, 4.94)	0.73 (0.12, 4.00)	1.06 (0.32, 3.43)	——
GP	**0.65 (0.43, 0.93)**	**0.32 (0.20, 0.52)**	**2.40 (1.27, 4.70)**	1.35 (0.74, 2.43)	1.25 (0.57, 2.72)	**1.55 (0.53, 4.60)**
KAI+GP vs						
KLTI+GP	1.02 (0.62, 1.68)	0.93 (0.50, 1.75)	1.12 (0.47, 2.54)	1.38 (0.59, 3.28)	0.44 (0.11, 1.66)	——
SMI+GP	0.76 (0.43, 1.34)	0.94 (0.48, 1.92)	**0.43 (0.17, 1.04)**	1.10 (0.45, 2.81)	**0.20 (0.05, 0.79)**	——
SQFZI+GP	0.86 (0.51, 1.49)	0.97 (0.50, 1.88)	0.57 (0.22, 1.45)	2.47 (0.88, 6.77)	0.67 (0.18, 2.50)	——
XAPI+GP	0.78 (0.35, 1.86)	0.99 (0.24, 4.38)	1.27 (0.41, 3.81)	1.57 (0.24, 9.25)	1.04 (0.23, 4.72)	——
GP	**0.54 (0.36, 0.82)**	**0.30 (0.18, 0.48)**	1.86 (0.89, 3.71)	**2.88 (1.33, 6.32)**	1.25 (0.36, 4.15)	——
KLTI+GP vs						
SMI+GP	0.74 (0.46, 1.20)	1.02 (0.53, 1.92)	**0.38 (0.19, 0.78)**	0.80 (0.44, 1.45)	0.46 (0.19, 1.10)	1.75 (0.15, 16.8)
SQFZI+GP	0.84 (0.54, 1.33)	1.04 (0.57, 1.87)	0.51 (0.24, 1.09)	1.78 (0.84, 3.84)	1.52 (0.67, 3.50)	1.66 (0.44, 6.62)
XAPI+GP	0.77 (0.36, 1.67)	1.06 (0.28, 4.66)	1.13 (0.43, 3.03)	1.14 (0.20, 5.51)	2.39 (0.79, 6.92)	——
GP	**0.53 (0.40, 0.70)**	**0.32 (0.21, 0.46)**	**1.66 (1.07, 2.58)**	**2.09 (1.43, 3.05)**	**2.82 (1.60, 5.21)**	**2.86 (1.20, 7.28)**
SMI+GP vs						
SQFZI+GP	1.14 (0.67, 1.88)	1.02 (0.52, 2.02)	1.34 (0.58, 3.07)	2.22 (0.99, 5.20)	**3.29 (1.37, 8.00)**	0.95 (0.10, 11.89)
XAPI+GP	1.02 (0.46, 2.35)	1.04 (0.26, 4.73)	**2.98 (1.06, 8.43)**	1.42 (0.24, 7.30)	**5.14 (1.69, 15.65)**	——
GP	0.72 (0.48, 1.04)	**0.31 (0.19, 0.52)**	**4.35 (2.49, 7.71)**	**2.61 (1.67, 4.16)**	**6.09 (3.24, 12.3)**	1.64 (0.22, 16.33)
SQFZI+GP vs						
XAPI+GP	0.91 (0.41, 2.09)	1.02 (0.25, 4.50)	2.23 (0.75, 6.48)	0.64 (0.11, 3.49)	1.57 (0.53, 4.54)	——
GP	**0.63 (0.45, 0.87)**	**0.31 (0.20, 0.47)**	**3.25 (1.76, 6.11)**	1.17 (0.59, 2.32)	**1.86 (1.05, 3.45)**	1.71 (0.60, 4.85)
XAPI+GP vs						
GP	0.7 (0.33, 1.41)	0.30 (0.07, 1.11)	1.46 (0.61, 3.48)	1.84 (0.39, 10.08)	1.19 (0.49, 3.03)	——

Note: The differences between the compared groups were deemed as significant when the 95% CI of the OR did not contain 1.00, which is underlined and bold. ADI, Aidi injection; CKSI, Compound Kushen injection; DLSI, Delisheng injection; GP, Gemcitabine plus Cisplatin; JOEI, Javanica oil emulsion injection; KAI, Kangai injection; KLTI, Kanglaite injection; SMI, Shenmai injection; SQFZI, Shenqifuzheng injection; XAPI, Xiaoaiping injection.

**FIGURE 5:**
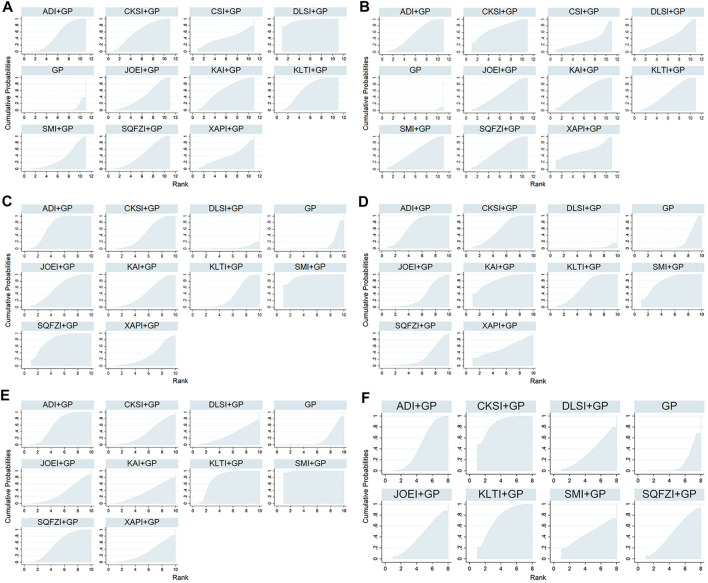
Surface under the cumulative ranking curves for outcomes. **(A)** Clinical effectiveness rate; **(B)** Performance status; **(C)** Leukopenia; **(D)** Gastrointestinal reactions; **(E)** Thrombocytopenia; and **(F)** Abnormal liver function. Note: ADI, Aidi injection; CKSI, Compound Kushen injection; DLSI, Delisheng injection; GP, Gemcitabine plus Cisplatin; JOEI, Javanica oil emulsion injection; KAI, Kangai injection; KLTI, Kanglaite injection; SMI, Shenmai injection; SQFZI, Shenqifuzheng injection; and XAPI, Xiaoaiping injection.

Based on the ranking result of the clinical effectiveness rate, the relative ranking of interventions for improving the clinical effectiveness rate was as follows: Delisheng injection + GP (94.6%) > Kanglaite injection + GP (67.43%) > Compound kushen injection + GP (63.92%) > Kangai injection + GP (63.31%) > Aidi injection + GP (50.99%) > Shenqifuzheng injection + GP (46.34%) > Javanica oil emulsion injection + GP (43.67%) > Chansu injection + GP (43.22%) > Xiaoaiping injection + GP (39.36%) > Shenmai injection + GP (32.59%) > GP only (4.564%). The results of ranking probabilities based on SUCRA are shown in Figure 5A and Table 3.

**TABLE 3:** Ranking probability of the various interventions among all interventions.

Interventions	Clinical effectiveness rate		Performance status		Leukopenia		Gastrointestinal reactions		Thrombocytopenia		Abnormal liver function	
	SUCRA(%)	Rank	SUCRA (%)	Rank	SUCRA (%)	Rank	SUCRA (%)	Rank	SUCRA (%)	Rank	SUCRA (%)	Rank
ADI+GP	50.99	5	56.47	5	74.97	3	71.12	3	64.98	3	48.22	4
CKSI+GP	63.92	3	73.36	1	49.47	5	61.12	5	38.82	5	87.17	1
CSI+GP	43.22	8	35.31	10	-	-	-	-	-	-	-	-
DLSI+GP	94.6	1	45.86	9	3.91	10	2.456	10	32.84	8	35.93	7
JOEI+GP	43.67	7	52.34	8	63.47	4	35.85	7	34.96	7	43.39	6
KAI+GP	63.31	4	61.13	2	46.85	6	82.62	1	36.46	6	-	-
KLTI+GP	67.43	2	53.65	7	38.99	7	63.89	4	81.92	2	75.31	2
SMI+GP	32.59	10	55.71	6	94.59	1	80.69	2	99.18	1	46.97	5
SQFZI+GP	46.34	6	57.89	3	81.25	2	28.65	8	60.08	4	48.45	3
XAPI+GP	39.36	9	57.29	4	34.25	8	55.13	6	32.71	9	-	-
GP	4.564	11	0.9969	11	12.24	9	18.48	9	18.06	10	14.55	8

Note: ADI, Aidi injection; CKSI, Compound Kushen injection; DLSI, Delisheng injection; GP, Gemcitabine plus Cisplatin; JOEI, Javanica oil emulsion injection; KAI, Kangai injection; KLTI, Kanglaite injection; SMI, Shenmai injection; SQFZI, Shenqifuzheng injection; XAPI, Xiaoaiping injection.

#### Performance Status

A total of 40 studies with 10 CHIs and 11 interventions reported performance status in the NMA. The results suggested that 8 types of CHIs: Aidi injection, Compound kushen injection, Delisheng injection, Javanica oil emulsion injection, Kangai injection, Kanglaite injection, Shenmai injection, and Shenqifuzheng injection, combined with GP were significantly more effective than GP alone. There were no statistically significant differences between the other interventions. The network graph is depicted in Figure 3B, and the ORs with 95% CI are presented in Table 2.

Based on the ranking result of performance status, the relative ranking of the interventions to improve performance status was as follows: Compound kushen injection + GP (73.36%) > Kangai injection + GP (61.13%) > Shenqifuzheng injection + GP (57.89%) > Xiaoaiping injection + GP (57.29%) > Aidi injection + GP (54.47%) > Shenmai injection + GP (55.71%) > Kanglaite injection + GP (53.65%) > Javanica oil emulsion injection + GP (52.34%) > Delisheng injection + GP (45.86%) > Chansu injection + GP (35.31%) > GP only (0.9969%). The results of the ranking probabilities based on SUCRA are shown in Figure 5B and Table 3.

#### Leukopenia

A total of 50 studies with 10 CHIs and 11 interventions reported leukopenia in the NMA. The results suggested that 6 types of CHIs: Aidi injection, Compound kushen injection, Javanica oil emulsion injection, Kanglaite injection, Shenmai injection, and Shenqifuzheng injection, combined with GP were significantly more effective than GP alone. Combined with GP, Aidi injection might have a more significant potential to reduce leukopenia than Delisheng injection and Kanglaite injection. In addition, Compound kushen injection combined with GP could have greater potential to reduce the leukopenia than Delisheng injection; Shenmai injection was better than Compoun kushen injection, Delisheng injection, Kangai injection, Kanglaite injection and Xiaoaiping injection; Javanica oil emulsion injection was better than Shenqifuzheng injection and Delisheng injection. There were no statistically significant differences between the other interventions. The network graph is depicted in Figure 3C, and the ORs with 95% CI are presented in Table 2.

Based on the ranking result of leukopenia, the relative ranking of the interventions to reduce leukopenia was as follows: Shenmai injection + GP (94.59%) > Shenqifuzheng injection + GP (81.25%) > Aidi injection + GP (74.97%) > Javanica oil emulsion injection + GP (63.47%) > Compound kushen injection + GP (49.47%) > Kangai injection + GP (46.85%) > Kanglaite injection + GP (38.99%) > Xiaoaiping injection + GP (34.25%) > GP only (12.24%) > Delisheng injection + GP only (3.91%). The results of the ranking probabilities based on SUCRA are shown in Figure 5C and Table 3.

#### Gastrointestinal Reactions

A total of 43 studies with 9 CHIs and 10 interventions reported gastrointestinal reactions in the NMA. The results suggested that 3 types of CHIs: Aidi injection, Compound kushen injection, Kangai injection, Kanglaite injection and Shenmai injection, combined with GP were significantly more effective than GP alone. In combination with GP, Aidi injection, Compound kushen injection, Kangai injection, Kanglaite injection, and Shenmai injection could have greater potential to reduce the gastrointestinal reactions than Delisheng injection. The network graph is depicted in Figure 3D, and the ORs with 95% CI are presented in Table 2.

Based on the ranking result of gastrointestinal reactions, the relative ranking of interventions to reduce the gastrointestinal reactions was as follows: Kangai injection + GP (82.62%) > Shenmai injection + GP (80.69%) > Aidi injection + GP (71.12%) > Kanglaite injection + GP (63.89%) > Compound kushen injection + GP (61.12%) > Xiaoaiping injection + GP (55.13%) > Javanica oil emulsion injection + GP (35.85%) > Shenqifuzheng injection + GP (28.65%) > GP only (18.48%) > Delisheng injection + GP (2.456%). The results of the ranking probabilities based on SUCRA are shown in Figure 5D and Table 3.

#### Thrombocytopenia

A total of 33 studies with 9 CHIs and 10 interventions reported thrombocytopenia in the NMA. The results suggested that 3 types of CHIs: Aidi injection, Kanglaite injection, and Shenqifuzheng injection, combined with GP, were significantly more effective than GP alone. Combined with GP, Shenmai injection might have a greater potential to reduce the thrombocytopenia than Aidi injection, Compoun kushen injection, Delisheng injection, Javanica oil emulsion injection, Kangai injection, Shenqifuzheng injection and Xiaoaiping injection. The network graph is depicted in Figure 3E, and the ORs with 95% CI are presented in Table 2.

Based on the ranking result of thrombocytopenia, the relative ranking of interventions to reduce thrombocytopenia was as follows: Shenmai injection + GP (99.18%) > Kanglaite injection + GP (81.92%) > Aidi injection + GP (64.98%) > Shenqifuzheng injection + GP (60.08%) > Compound kushen injection + GP (38.82%) > Kangai injection + GP (36.46%) > Javanica oil emulsion injection + GP (34.96%) > Delisheng injection + GP (32.84%) > Xiaoaiping injection + GP (32.71%) > GP only (18.06%). The results of the ranking probabilities based on SUCRA are shown in Figure 5E and Table 3.

#### Abnormal Liver Function

A total of 20 studies with 7 CHIs and 8 interventions reported abnormal liver function in the NMA. The results suggested that 4 types of CHIs: Aidi injection, Compound kushen injection, Javanica oil emulsion injection, and Kanglaite injection, combined with GP, were significantly more effective than GP alone. There were no statistically significant differences between the other interventions. The network graph is depicted in Figure 3F and the ORs with 95% CI are presented in Table 2.

Based on the ranking result of abnormal liver function, the relative ranking of interventions to reduce abnormal liver function was as follows: Compound kushen injection + GP (87.17%) > Kanglaite injection + GP (75.31%) > Shenqifuzheng injection + GP (48.45%) > Aidi injection + GP (48.22%) > Shenmai injection + GP (46.97%) > Javanica oil emulsion injection + GP (43.39%) > Delisheng injection + GP (35.93%) > GP only (14.55%). The results of the ranking probabilities based on SUCRA are shown in Figure 5F and Table 3.

### Cluster Analysis

**FIGURE 6:**
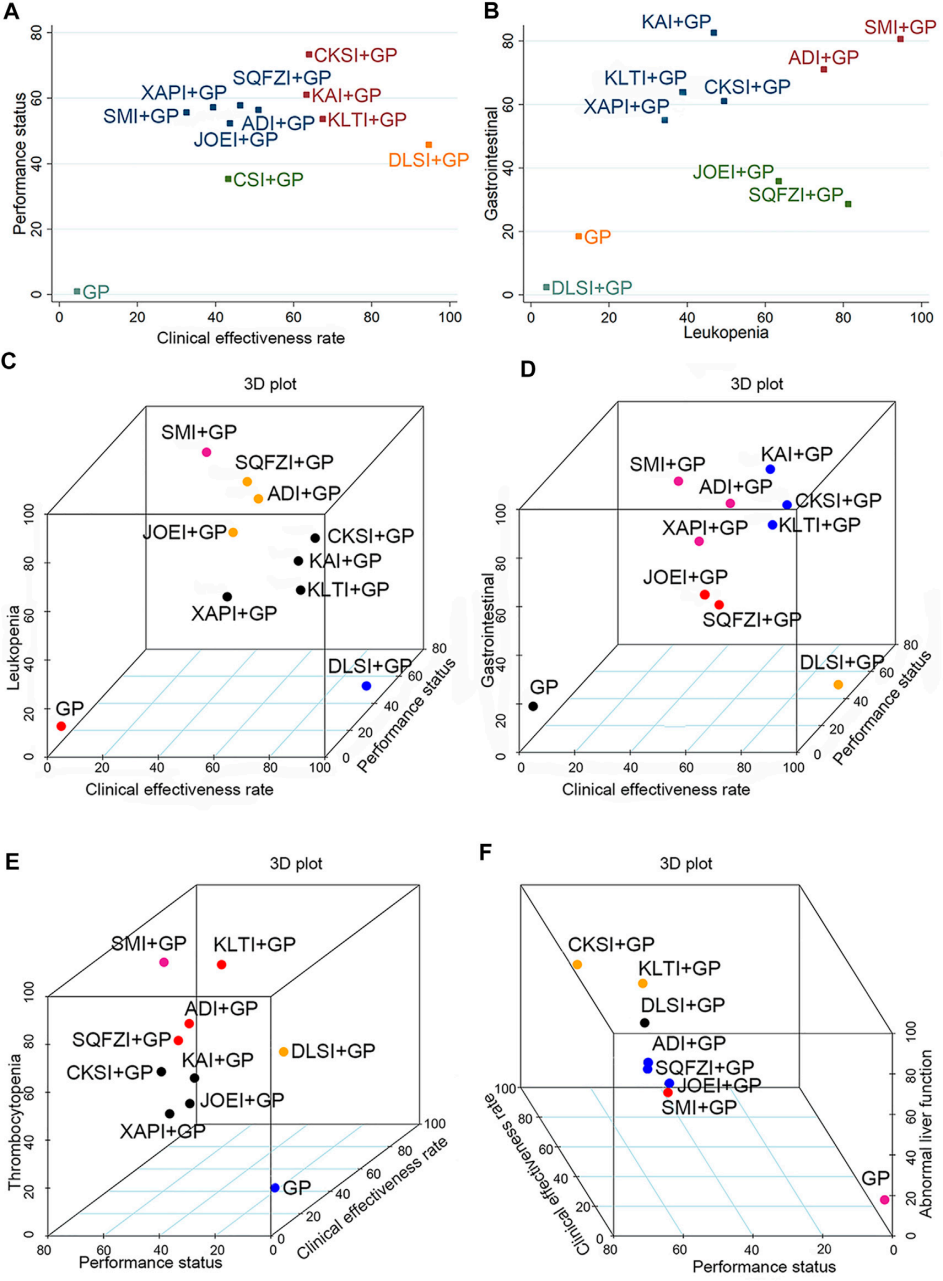
Cluster analysis plots for six outcomes. **(A)** Clinical effectiveness rate (x-axis) and performance status (y-axis); **(B)** Leukopenia (x-axis) and gastrointestinal reactions (y-axis); **(C)** Clinical effectiveness rate (x-axis), performance status (y-axis), and leukopenia (z-axis); **(D)** Clinical effectiveness rate (x-axis), performance status (y-axis), and gastrointestinal reactions (z-axis); **(E)** Performance status (x-axis), clinical effectiveness rate (y-axis), and thrombocytopenia (z-axis); **(F)** Performance status (x-axis), clinical effectiveness rate (y-axis), and abnormal liver function (z-axis). Note: Interventions with the same color belong to the same cluster, and interventions located in the upper-right corner indicate optimal therapy for two different outcomes. ADI, Aidi injection; CKSI, Compound Kushen injection; DLSI, Delisheng injection; GP, Gemcitabine plus Cisplatin; JOEI, Javanica oil emulsion injection; KAI, Kangai injection; KLTI, Kanglaite injection; SMI, Shenmai injection; SQFZI, Shenqifuzheng injection; and XAPI, Xiaoaiping injection.

Cluster analysis was used to comprehensively compare the effects of the interventions on two different outcomes. Eight interventions reported both clinical effectiveness rate and performance status. Compared with other interventions, Compound kushen injection + GP, Kangai injection + GP, and Kanglaite injection + GP were similarly superior, and GP alone produced the worst result. Moreover, in terms of reducing leukopenia and gastrointestinal reactions in 10 interventions, Shenmai injection + GP and Aidi injection + GP showed the most favorable benefit. At the same time, GP alone yielded the worst result. Different colored dots indicate different types of interventions in Figure 6.

When cluster analysis was conducted on 10 interventions reporting clinical effectiveness rate, performance status, and leukopenia, Shenmai injection + GP had the highest probability. In contrast, GP only had the worst ranking result. Similarly, for clinical effectiveness rate, performance status and thrombocytopenia, Shenmai injection + GP had the highest probability among the 8 interventions. Moreover, in the comprehensive ranking of clinical effectiveness rate, performance status, and gastrointestinal reactions among 10 interventions, Compound kushen injection + GP, Kangai injection + GP and Kanglaite injection + GP had advantages in the ranking, while GP only yielded the worst result. In addition, Compound kushen injection + GP and Kanglaite injection + GP had advantages in ranking, while GP only yielded the worst result in terms of clinical effectiveness rate, performance status, and abnormal liver function. Different colored dots indicate different types of interventions in Figure 6.

### Publication Bias

**FIGURE 7:**
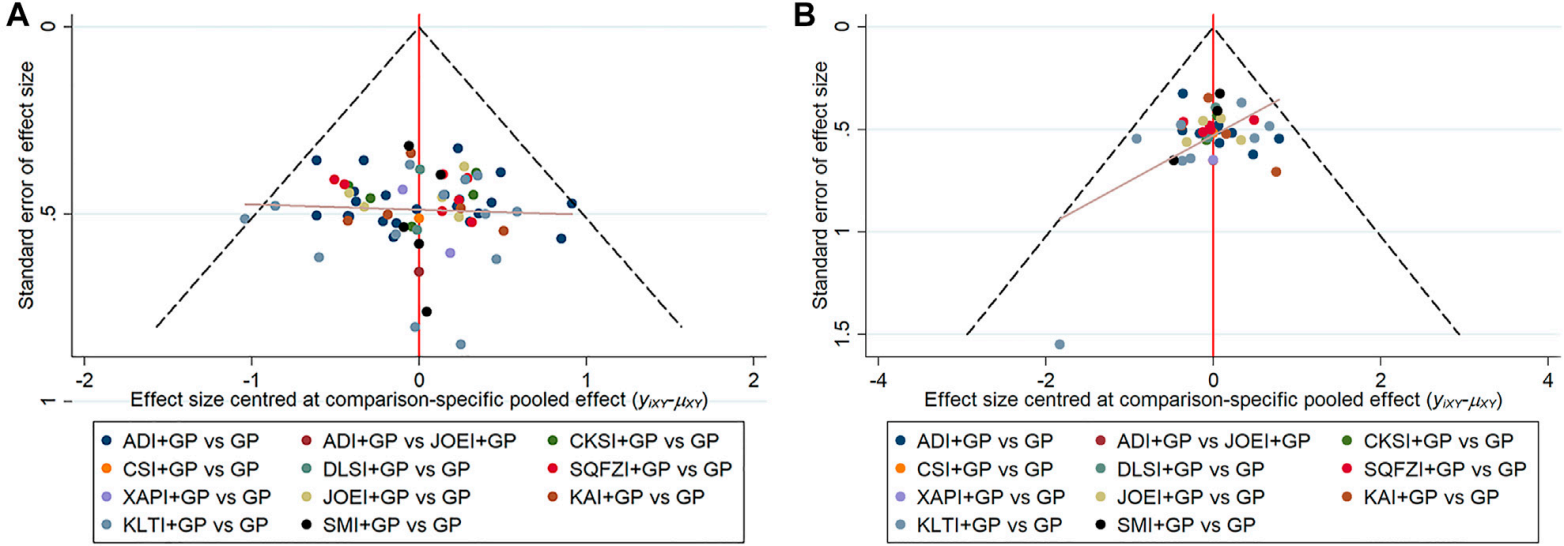
Comparison-adjusted funnel plot for outcomes. **(A)** clinical effectiveness rate; **(B)** performance status. Note: ADI, Aidi injection; CKSI, Compound Kushen injection; DLSI, Delisheng injection; GP, Gemcitabine plus Cisplatin; JOEI, Javanica oil emulsion injection; KAI, Kangai injection; KLTI, Kanglaite injection; SMI, Shenmai injection; SQFZI, Shenqifuzheng injection; and XAPI, Xiaoaiping injection.

Comparison-adjusted funnel plots for the clinical effectiveness rate and performance status were used to test for publication bias. As shown in Figure 7 of the clinical effectiveness rate and performance status, the determined angles between the correction auxiliary line and the centerline indicated that this study had potential publication bias.

## Discussion

To compare the efficacy outcomes of different CHIs in combination with GP for NSCLC, this study used the NMA method to analyze evidence-based data from RCTs. Based on the results of NMA, Delisheng injection in combination with GP was the best choice for improving short-term clinical efficacy in patients. In terms of improvement in performance status and reduction in abnormal liver function, Compound kushen injection in combination with GP performed significantly better than the other injections. In terms of leukopenia and thrombocytopenia, Shenmai injection was the best. In addition, Kangai injection in combination with GP had the best effect on reducing gastrointestinal reactions. However, clinicians should choose different treatments based on patients' specific conditions.

With a poor prognosis, NSCLC is the leading cause of cancer death and seriously threatens human health. GP regimen is commonly used in the treatment of NSCLC patients, and highly toxic drug reactions can even lead to treatment discontinuation and failure. As a complementary and alternative medicine, TCM for lung cancer, especially advanced lung cancer, has accumulated rich clinical experience, which can improve patients' quality of life and long-term survival. Shenmai injection is mainly prepared from the extract of *Panax ginseng* C.A.Mey. [Araliaceae; Ginseng radix et rhizome], *Ophiopogon japonicus* (Thunb.) Ker Gawl. [Liliaceae; Ophiopogonis radix]. A study (Sun et al., 2020) have shown that Shenmai injection can reprogram glucose metabolism and enhance the sensitivity of lung cancer cells to chemotherapy drugs through the AKT/mTOR/c-Myc pathway. Ginsenoside Rg3, the main component of Shenmai injection, could decrease chemoresistance-induced expression of the programmed death ligand 1, increase immunomodulatory actions and response to DNA damage, and thereby inhibit tumorigenesis and viability of lung cancer cells (Park et al., 2011; Jiang et al., 2017; Liu et al., 2019). Compound Kushen injection is mainly prepared by extracting *Sophora flavescens* Aiton [Fabaceae; Sophorae flavescentis radix] and *Heterosmilax yunnanensis* Gagnep. [Liliaceae]. Moreover, the main components of Compound Kushen injection are matrine and oxymatrine, which can inhibit the growth of tumor cells (Pu et al., 2018).

There has been only one previous NMA of different CHIs combined with GP for NSCLC, which was published in 2014 and included 12 CHIs (Tian et al., 2014). In contrast, this NMA has the following merits. First, the NMA used multidimensional cluster analysis for the first time to visualize the result of three outcomes comprehensively. Second, ADRs such as thrombocytopenia and abnormal liver function were added as outcome indicators for patients. The cluster analysis was performed based on the SUCRA values to select the superior CHIs in terms of efficacy. Finally, the eligibility criteria in this NMA were strictly formulated and defined. In particular, the patients of the included RCTs were restricted to stage Ⅲ or Ⅳ NSCLC to reduce clinical heterogeneity. In that study, Aidi injection, Kangai injection, and Compound Kushen injection ranked ahead in terms of leukopenia and gastrointestinal reactions. Excluding the different CHIs of the two network researches, the CHIs had a similar ranking for these two outcomes.

However, the limitations of the current NMA cannot be avoided. First, 21 (29.58%) studies adequately reported the random sequence generation methods, while none of the included studies mentioned detailed information about allocation concealment and blinding methods, which may lead to selection bias, performance bias and detection bias. Second, most interventions of the included studies were CHIs in combination with GP versus GP, and only 1 study directly compared the efficacy of two Chinese herbal injections, which is a small proportion. Finally, limited by the application scope of CHIs, all studies were conducted in China, and all patients were Chinese. Therefore, the results may not be generalizable.

## Conclusion

Current evidence revealed that CHIs combined with GP have a better impact on patients with NSCLC than GP alone. Aidi injection, Compound kushen injection, and Kanglaite injection deserve more attention of clinicians when combined with GP in patients with NSCLC. Additionally, due to the limitations of this NMA, more head-to-head RCTs are needed to properly support our findings.

## Abbreviations

95% CI, 95% Credible intervals; ADRs, Adverse drug reactions; CHIs, Chinese herbal injections; CR, complete response; IF, Inconsistency factors; KPS, Karnofsky performance status; MeSH, Medical subject heading; NMA, Network meta-analysis; NSCLC, Non-small-cell lung cancer; OR, Odds ratio; PD, Progressive disease; PR, Partial response; PRISMA, the preferred reporting items for systematic reviews and meta-analyses; RCTs, Randomized controlled trials; REML, Restricted maximum likelihood; SD, Stable disease; SUCRA, the surface under the cumulative ranking; TNM, Tumor node metastasis.
